# Antigen-Dependent Inducible T-Cell Reporter System for PET Imaging of Breast Cancer and Glioblastoma

**DOI:** 10.2967/jnumed.122.264284

**Published:** 2023-01

**Authors:** Jaehoon Shin, Matthew F.L. Parker, Iowis Zhu, Aryn Alanizi, Carlos I. Rodriguez, Raymond Liu, Payal B. Watchmaker, Mausam Kalita, Joseph Blecha, Justin Luu, Brian Wright, Suzanne E. Lapi, Robert R. Flavell, Hideho Okada, Thea D. Tlsty, Kole T. Roybal, David M. Wilson

**Affiliations:** 1 Department of Radiology and Biomedical Imaging, University of California, San Francisco, San Francisco, California;; 2 Department of Microbiology and Immunology, University of California, San Francisco, San Francisco, California;; 3 Parker Institute for Cancer Immunotherapy, San Francisco, California;; 4 Department of Pathology, University of California, San Francisco, San Francisco, California;; 5 Department of Neurological Surgery, University of California, San Francisco, San Francisco, California;; 6 Department of Radiology, University of Alabama at Birmingham, Birmingham, Alabama;; 7 Helen Diller Cancer Center, University of California, San Francisco, San Francisco, California;; 8 Chan Zuckerberg Biohub, San Francisco, California;; 9 Gladstone UCSF Institute for Genetic Immunology, San Francisco, California; and; 10 UCSF Cell Design Institute, San Francisco, California

**Keywords:** SNIPR, CAR T, PET, reporter, cancer antigens

## Abstract

For the past several decades, chimeric antigen receptor T-cell therapies have shown promise in the treatment of cancers. These treatments would greatly benefit from companion imaging biomarkers to follow the trafficking of T cells in vivo. **Methods:** Using synthetic biology, we engineered T cells with a chimeric receptor synthetic intramembrane proteolysis receptor (SNIPR) that induces overexpression of an exogenous reporter gene cassette on recognition of specific tumor markers. We then applied a SNIPR-based PET reporter system to 2 cancer-relevant antigens, human epidermal growth factor receptor 2 (HER2) and epidermal growth factor receptor variant III (EGFRvIII), commonly expressed in breast and glial tumors, respectively. **Results:** Antigen-specific reporter induction of the SNIPR PET T cells was confirmed in vitro using green fluorescent protein fluorescence, luciferase luminescence, and the HSV-TK PET reporter with 9-(4-^18^F-fluoro-3-[hydroxymethyl]butyl)guanine ([^18^F]FHBG). T cells associated with their target antigens were successfully imaged using PET in dual-xenograft HER2+/HER2− and EGFRvIII+/EGFRvIII− animal models, with more than 10-fold higher [^18^F]FHBG signals seen in antigen-expressing tumors versus the corresponding controls. **Conclusion:** The main innovation found in this work was PET detection of T cells via specific antigen-induced signals, in contrast to reporter systems relying on constitutive gene expression.

## Visual Abstract

**Figure:**
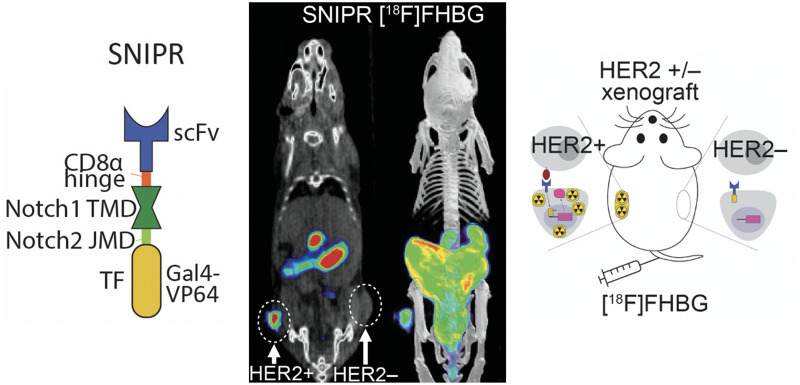

Chimeric antigen receptor T-cell (CAR T) therapy has revolutionized oncology, demonstrating promising results for refractory drug-resistant leukemias and lymphomas (*1*–*3*). among other cancers. CAR T cells are engineered to respond to cancer cells expressing a specific protein target, inducing rapid cell division and clonal expansion within the tumor microenvironment and activating immune response to target cells via local secretion of cytokines, interleukins, and growth factors (Supplemental Fig. 1A; supplemental materials are available at http://jnm.snmjournals.org) (*4**,**5*). Hundreds of clinical trials have been initiated globally to study CAR T cells, with 2 of the most popular targets being CD19 (*6*) and B-cell maturation antigen (seen in multiple myeloma) (*7**,**8*). Importantly, there were engineered T-cell clinical trials resulting in patient deaths due to off-target effects, which may have been mediated by recognition of normal lung (*9*) or cardiac (*10**,**11*) tissues. We currently do not have tools to detect the engineered T cells engaging antigens in off-target tissues in vivo before advanced tissue damage, which can be identified only via biopsy or autopsy. Noninvasive methods are therefore critical in evaluating the safety of preclinical CAR T therapies and CAR T clinical trials by providing surrogate real-time maps for patient-specific T-cell–target antigen interactions.

Current limitations in predicting CAR T safety in vivo are potentially addressed by the PET-compatible synthetic intramembrane proteolysis receptor (SNIPR) T cells described in this article. The SNIPR system has produced a powerful new class of chimeric receptors that bind to target surface antigens and induce transcription of exogenous reporter genes via release of a transcription factor domain by regulated intramembrane proteolysis (Supplemental Fig. 1B). Importantly, SNIPRs can be designed to have an identical single-chain variable fragment (scFv) domain as CARs, thereby providing a surrogate map for CAR–antigen interaction. The SNIPR contains the regulatory transmembrane domain of human Notch receptor but bears an extracellular antigen recognition domain (e.g., scFv) and an intracellular transcriptional activator domain (Gal4-VP64). When the SNIPR engages its target antigen on an opposing cell, intramembrane cleavage is induced, releasing the intracellular transcriptional domain Gal4 and allowing it to enter the nucleus to activate transcription of target genes (*12**,**13*).

Despite the great versatility of synNotch and SNIPR, their diagnostic potential has not yet been explored. Current PET approaches producing antigen-dependent signals are dominated by immuno-PET, whereby a monoclonal antibody is labeled with a radioisotope such as [^89^Zr] (*14*–*16*). In the described SNIPR approach, PET signals also depend on the interaction between an antigen and its corresponding scFv but occur via T-cell–based overexpression of the HSV-TK reporter. In this study, we combined CAR and SNIPR technologies to develop a new T-cell–based molecular sensor that can image T cells engaged with their target antigens. On binding an antigen target, CAR produces rapid T-cell division within the tumor microenvironment, and SNIPR activates the overexpression of PET imaging reporter genes. We developed human epidermal growth factor receptor 2 (HER2) and epidermal growth factor receptor variant III (EGFRvIII)–specific SNIPR T cells that were successfully imaged in vivo on interaction with their corresponding antigen-expressing tumors. We also compared HER2-specific SNIPR T cells with the naked [^89^Zr]-modified anti-HER2 monoclonal antibody ([^89^Zr]trastuzumab) used in immuno-PET to validate the specificity of the cell-based method. This proof-of-concept study provides the foundation for applying the SNIPR PET reporter to high-sensitivity cell-based antigen detection as well as mapping of engineered T-cell–antigen interactions in vivo.

## MATERIALS AND METHODS

The supplemental materials provide detailed information on molecular biology, radiosynthesis, and several imaging studies not reported in the main text.

### Receptor and Response Element Construct Design

#### SNIPR and CAR Design

A full description of SNIPR and CAR receptors, including variable-affinity scFvs, can be found in the supplemental materials (*17**,**18*). SNIPRs were built by fusing anti-HER-2 scFvs or anti-EGFRvIII scFvs with a truncated CD8α hinge region (TTTPAPRPPTPAPTIASQPLSLRPEAC), human Notch1 transmembrane domain (FMYVAAAAFVLLFFVGCGVLLS), intracellular Notch2 juxtamembrane domain (KRKRKH), and Gal4-VP64 transcriptional element. CARs were built by fusing a binding head (anti-HER2 4D5-8 scFv or interleukin 13 [IL13] mutein), CD8ɑ transmembrane domain, costimulatory domain 4-1BB, CD3ζ, and enhanced green fluorescent protein (eGFP).

#### Reporter Design

The reporter constructs used are fully described in the supplemental materials. All reporter constructs were cloned into either a modified pHR’SIN:CSW vector containing a Gal4-upstream activation sequence response element with CMV (Gal4UAS-RE-CMV) promoter followed by multiple-cloning-site pGK promoter and mCherry or a modified pHR’SIN:CSW vector containing a Gal4UAS-RE-CMV promoter followed by multiple-cloning-site 3-phosphoglycerate kinase (pGK)-promoter and mCitrine. The HSV-derived thymidine kinase with SR39 mutation and GFP fusion (HSV-TKSR39-GFP) construct was cloned from HSV-thymidine kinase-GFP fusion gene with eukaryotic translation elongation factor 1α promoter (cEF.TK-GFP) (plasmid 33308; Addgene), which was deposited by Pomper et al. (*19*) using site-directed mutagenesis as described in Supplemental Figure 2A. The HSV-TKSR39-T2A-interleukin 2 superkine (sIL2) construct was cloned from HSV-TKSR39-GFP using In-Fusion cloning (Takara Bio) after adding 6 C-terminal amino acids (EMGEAN) that were deleted in the original HSV-TK in cEF.TK-GFP, as shown in Supplemental Figure 2B.

### Preparation of SNIPR T Cells

#### Primary Human T-Cell Isolation and Culture

A full description of T-cell isolation and culture is presented in the supplemental materials. Primary CD4+ and CD8+ T cells were isolated by negative selection (catalog nos. 15062 and 15063; STEMCELL Technologies). CD4+ T cells and CD8+ T cells were separated using a Biolegend MojoSort human CD4 T-cell isolation kit (catalog no. 480130; Biolegend) following the manufacturer’s protocol. For experiments involving the induction of interleukin 2 (IL2) superkine, primary T cells were maintained in human T-cell medium supplemented with IL2 until experimentation, whereupon medium was replaced with medium without added IL2.

#### Lentiviral Transduction of Human T Cells

Human T cells were transduced as described in the supplemental materials. Pantropic VSV-G pseudotyped lentivirus was produced via transfection of Lenti-X 293T cells (catalog no. 11131D; Clontech) with a pHR’SIN:CSW transgene expression vector and the viral packaging plasmids pCMVdR8.91 and pMD2.G using TransIT-Lenti (catalog no. MIR6606; Mirus). Primary T cells were thawed the same day, and after 24 h in culture, they were stimulated with human T-activator CD3/CD28 Dynabeads (catalog no. 11131D; Life Technologies) at a 1:3 cell-to-bead ratio. At 48 h, viral supernatant was harvested, and the primary T cells were exposed to the virus for 24 h. At day 5 after T-cell stimulation, the CD3/CD28 Dynabeads were removed, and the T cells were sorted for assays with a Beckton Dickinson (BD) FACSAria II.

#### SNIPR T-Cell Design for In Vivo Imaging

For HER2+ and HER2− xenograft bioluminescence imaging, we generated anti-HER2 SNIPR T cells with 3 constructs: constitutively expressed anti-HER2(4D5-8) SNIPR, constitutively expressed anti-HER2(4D5-8) CAR, and conditionally expressed firefly luciferase. At baseline, anti-HER2 SNIPR T cells express anti-HER2 SNIPR and anti-HER2 CAR. On binding to HER2, they overexpress fLuc through SNIPR activation and proliferate by CAR activation. For HER2+ and HER2− xenograft PET/CT imaging, we generated anti-HER2 SNIPR T cells with 3 constructs: constitutively expressed anti-HER2(4D5-8) SNIPR, constitutively expressed anti-HER2(4D5-8) CAR, and conditionally expressed HSV-TKSR39-T2A-sIL2. At baseline, anti-HER2 SNIPR T cells express anti-HER2 SNIPR and anti-HER2 CAR. On binding to HER2, they overexpress HSV-TKSR39 and IL2 superkine by SNIPR activation and also proliferate by CAR activation.

For EGFRvIII+ and EGFRvIII− xenograft bioluminescence imaging, we generated anti-EGFRvIII SNIPR T cells with 2 constructs: constitutively expressed anti-EGFRvIII(139) SNIPR and conditionally expressed IL13 mutein CAR-T2A-nanoLuc. At baseline, anti-EGFRvIII SNIPR T cells express anti-EGFRvIII SNIPR. On binding to EGFRvIII, they overexpress IL13 mutein CAR and nanoLuc. For EGFRvIII+ and EVFRvIII− xenograft bioluminescence imaging, we generated anti-EGFRvIII SNIPR T cells with 2 constructs: constitutively expressed anti-EGFRvIII(139) SNIPR and conditionally expressed anti-IL13m-CAR-HSV-TKSR39. At baseline, anti-EGFRvIII SNIPR T cells express anti-EGFRvIII SNIPR. On binding to EGFRvIII, they overexpress IL13 mutein CAR and HSV-TKSR39.

### In Vitro Studies

#### Cancer Cell Lines

The cell lines used were 293T (catalog no. CRL-3216; ATCC), MDA-MB-468 (catalog no. HTB-132, HER2− cells; ATCC), SKBR3 (catalog no. HTB-30, HER2+ high cells; ATCC), MCF7 (catalog no. HTB-22, HER2+ low cells; ATCC), U87-EGFRvIII–negative luciferase, and U87-EGFRvIII–positive luciferase (*12*). Culture media are further described in the supplemental materials.

#### In Vitro Fluorophore and Luciferase Reporter Assay

For in vitro SNIPR T-cell stimulations, 2 × 10^5^ T cells were cocultured with 1 × 10^5^ cancer cells and analyzed at 48–72 h for reporter expression. Production of fluorophores (GFP and cyan fluorescent protein) were assayed using flow cytometry with a BD LSR II, and the data were analyzed with FlowJo software (TreeStar). Production of firefly luciferase was assessed with the ONE-Glo luciferase assay system (catalog no. E6110; Promega), and production of nanoLuc luciferase was assessed with the Nano-Glo luciferase assay system (catalog no. N1110; Promega). Bioluminescence was measured with a FlexStation 3 (Molecular Devices).

#### In Vitro Radiotracer Uptake Assay

Radiosyntheses of 9-(4-^18^F-fluoro-3-[hydroxymethyl]butyl)guanine ([^18^F]FHBG) was performed using established techniques, summarized in Supplemental Figure 3 and the supplemental methods for radiosynthesis (*20*). For in vitro SNIPR T-cell stimulations, 1 × 10^6^ T cells were cocultured with 5 × 10^5^ cancer cells and analyzed at 48–72 h for radiotracer uptake. On the day of the radiotracer uptake experiment, T cells and cancer cells were resuspended and 74 kBq (2 μCi) of [^18^F]FHBG were added to each well and incubated for 3 h at 37°C, 5% CO_2_. After washing, retained radiotracer activity was measured using a Hidex γ-counter.

#### Reporter Assays with Varying Receptor Affinity and Abundance (Heat Map)

Heat maps for reporter expression were generated as described in the supplemental materials, with SNIPR receptors of varying HER2 binding affinities (4D5-3, 4D5-5, 4D5-7, and 4D5-8 scFv, in order of increasing binding affinity) and cancer cells with varying amounts of surface HER2 expression (293T, MCF7, and SKBR3, in order of increasing HER2 expression level).

### In Vivo Studies

#### Murine Models/Tumor Cohorts Studied

Both luciferase-based and PET reporter data were acquired, and 2 dual-xenograft models were studied. After determining the optimal time point using optical imaging, (8–10 d), PET imaging was performed at several time points, with killing of the animals to verify tissue tracer accumulation (γ-counting) and to perform histology and antigen staining (*21*–*23*).

#### Optical Imaging

As shown in Supplemental Figure 2, luciferase-based studies were performed initially (21 d) to investigate the optimal time point for [^18^F]FHBG detection of T-cell induction. The SNIPR T-cell distribution within tumor was determined by luminescence emission using a Xenogen IVIS Spectrum after intravenous d-luciferin injection according to the manufacturer’s directions (GoldBio).

#### PET Imaging

Radiosyntheses of [^18^F]FHBG, [^18^F]FDG, and [^89^Zr]trastzumab were performed as described in the supplemental materials (*20**,**24*). After radiotracer administration, mice were anesthetized under isoflurane, transferred to an Inveon small-animal PET/CT system (Siemens), and imaged using a single static 25-min PET acquisition followed by a 10-min small-animal CT scan for attenuation correction and anatomic coregistration.

#### [^18^F]FHBG SNIPR Model

Two murine models were studied: a dual HER2+/HER2− flank model (*n* = 7) and a dual EGFRvIII+/EGFRvIII− flank model (*n* = 4). For the HER2+/HER2–model, 1 million MD468 (HER2+ breast cancer cell line, fast-growing) and 3 million SKBR3 (HER2− breast cancer cell line, slow-growing) subcutaneously injected xenografts generated roughly similar-sized tumors in 3 wk. Therefore, 4 × 10^6^ SKBR3 cells and 1 × 10^6^ MD468 cells were implanted subcutaneously into 6- to 10-wk-old female NCG mice (Charles River). For the EGFRvIII+/EGFRvIII– model, 1 × 10^6^ EGFRvIII+ or EGFRvIII– U87 cells were implanted subcutaneously into 6- to 10-wk-old female NCG mice. All mice were then injected with 6.0 × 10^6^ SNIPR T cells via the tail vein in 100 μL of PBS. For PET imaging, 5,550 MBq (150 mCi) of [^18^F]FHBG were administered via the tail vein. One hour after injection, images were acquired at days 3, 6, 8, and 10 after T-cell injection. On completion of imaging at day 10, the mice were killed, and biodistribution analysis was performed. Harvested tissues were γ-counted using a Hidex automatic γ-counter.

#### [^18^F]FDG Model

The HER2+/HER2– model was developed as previously described; 4 × 10^6^ SKBR3 cells and 1 × 10^6^ MD468 cells were implanted subcutaneously into 6- to 10-wk-old female NCG mice, with 4 mice per group. For PET imaging, 5,550 MBq (150 mCi) of [^18^F]FDG were administered via the tail vein. At 1 h after injection, imaging was performed.

#### [^89^Zr]Trastuzumab Model

The same [^18^F]FDG cohort was used (*n* = 4) for the [^89^Zr]trastuzumab model. For PET imaging, 5,550 MBq (150 mCi) of [^89^Zr]trastuzumab were administered via the tail vein. At 3 d after injection, imaging was performed. On completion of imaging, the mice were killed, and biodistribution was analyzed. Harvested tissues were γ-counted using a Hidex automatic γ-counter.

### Data Analysis and Statistical Methods

Fluorescence-activated cell sorting analysis data were processed using FlowJo (BD Biosciences). All data graphs are depicted with error bars corresponding to the SEM. All statistical analyses of in vitro data were performed using Microsoft Excel, programming language R (https://www.R-project.org/), and Prism software (version 7.0; GraphPad). Data were analyzed using 1-way ANOVA or unpaired 2-tailed Student *t* tests. Small-animal PET/ CT data were analyzed using the open-source software AMIDE (*25*), and percentage injected dose (%ID) per volume was used for quantitative comparison. A 95% CI was used to distinguish significant differences in all cases.

## RESULTS

### Synthesis and In Vitro Validation of PET-Compatible Anti-HER2-SNIPR T Cells

**FIGURE 1.**
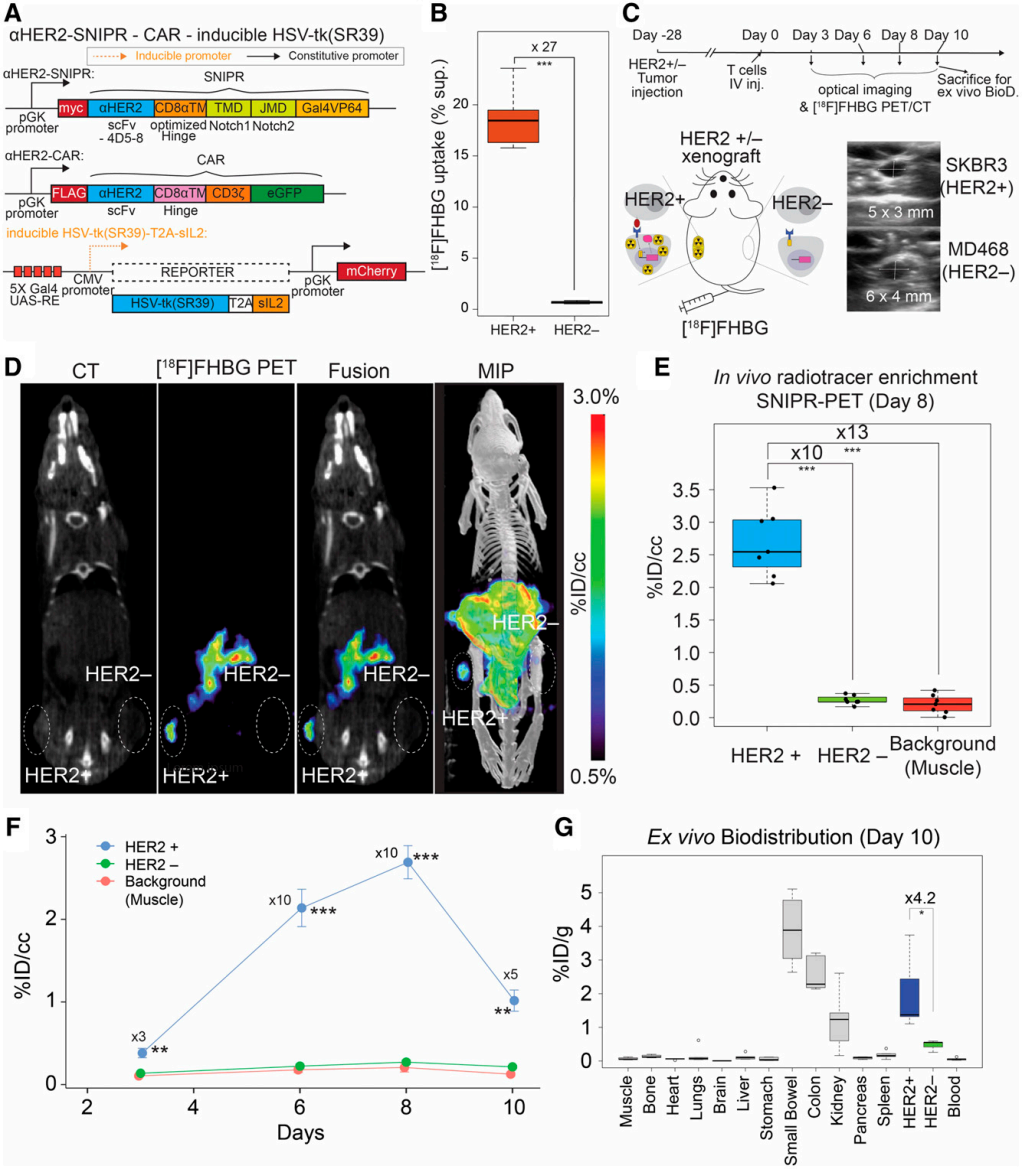
In vivo [^18^F]FHBG uptake in HER2+ tumors using activated anti-HER2 SNIPR T cells. (A) For PET/CT, we generated T cells by transducing 3 plasmids, including anti-HER2 SNIPR, anti-HER2 CAR, and inducible HSV-TK(SR39)-T2A-sIL2, followed by fluorescence-activated cell sorting using myc, GFP, and mCherry. (B) We repeated the in vitro radiotracer accumulation of activated SNIPR T cells also bearing CAR, following the experimental scheme shown in Supplemental Fig. 5B. We confirmed significantly higher [^18^F]FHBG accumulation in SNIPR T cells after coculturing with SKBR3 (HER2+) cells than in SNIPR T cells after coculturing with MB468 (HER2–) cells. (C) Double xenograft mouse models were generated by implanting SKBR3 (HER2+) and MD468 (HER2–) cells in left- and right-flank soft tissue. Four weeks after tumor implantation, SNIPR T cells were injected into tail veins. Small-animal PET/CT was performed 3, 6, 8, and 10 d after T-cell injection. (D) Representative CT images, [^18^F]FHBG PET images, [^18^F]FHBG PET/CT images, and maximum-intensity projection [^18^F]FHBG PET/CT images at day 8 demonstrated similar size of xenografts, with radiotracer enrichment only within SKBR3 (HER2+) xenograft and not within MD468 (HER2–) xenograft. (E) Quantitative ROI analyses of HER2+ and HER2– tumors and background (shoulder muscle) at day 8 demonstrated statistically significant radiotracer enrichment within HER2+ xenograft, 10 times and 13 times greater than within HER2– xenograft and background. (F) Time-dependent ROI analyses of radiotracer enrichment within HER2+ tumor demonstrated greatest radiotracer enrichment at day 8 after T-cell injection. Slightly decreased radiotracer enrichment was observed at day 10, at which point mice were killed for ex vivo analysis. (G) Biodistribution analysis (day 10) of [^18^F]FHBG enrichment within different organs demonstrated significantly greater [^18^F]FHBG enrichment within HER2+ xenograft than within HER2– xenograft. As seen on small-animal PET/CT, gastrointestinal system demonstrated high level of [^18^F]FHBG uptake. MIP = maximum-intensity projection. *P < 0.05. **P < 0.01. ***P < 0.001.

To investigate the SNIPR PET imaging approach, we started with one of the most extensively studied tumor antigens, HER2 (*26*). Following the published protocol by Roybal et al. (*26*), we transduced human CD4+ T cells with 2 plasmids encoding a SNIPR containing an anti-HER2 scFv (4D5-8), and an inducible reporter. For anti-HER2 SNIPR, we used the SNIPR with an anti-HER2 scFv binding head, an optimized truncated CD8α hinge region, the human Notch1 transmembrane domain, the intracellular Notch2 juxtamembrane domain, and a transcriptional element composed of Gal4-VP64 (Fig. 1A) (*27*). For the reporter, we used SR39 mutant herpes simplex virus–thymidine kinase (HSV1-sr39TK)–GFP fusion protein and enhanced firefly luciferase (fLuc) (*19**,**28**,**29*). In this study, we used the hyperactive mutant HSV1-sr39TK to maximize the detection sensitivity (Supplemental Fig. 2A) (*28**,**30*). When thymidine kinase (TK) reporter expression on SNIPR activation was being evaluated, HSV1-sr39TK-GFP fusion protein was applied instead for ready assessment using flow cytometry (Supplemental Fig. 1B). In all the subsequent radiotracer uptake experiments, however, HSV1-sr39TK was cloned with self-cleavage sequence T2A followed instead by IL2 superkine for a higher level of T-cell activation on SNIPR activation (Supplemental Fig. 1B).

We tested the induction of reporters, including inducible HSV1-sr39TK-GFP and inducible luciferase activity, in the anti-HER2 SNIPR system (Supplemental Fig. 4A). The newly synthesized anti-HER2 SNIPR T cells had transduction efficacy comparable to others we recently published (*26*) (Supplemental Fig. 4B). Anti-HER2 SNIPR T cells were cocultured with the HER2+ breast cancer line SKBR3 and the HER2– breast cancer line MD468 for 48 h to induce SNIPR activation and downstream reporter gene expression (Supplemental Fig. 4C). First, SNIPR activation induced over 160-fold expression of HSV1-sr39TK-GFP compared with negative control (*n* = 4, *P* < 0.001) (Supplemental Fig. 4D). Next, SNIPR activation induced over 30-fold higher luciferase activity than negative control (*n* = 4, *P* < 0.001) (Supplemental Fig. 4E). We also tested scFvs with varying binding affinities and cancer cells with varying antigen abundance (*n* = 3 per combination). As expected, HSV1-sr39TK-GFP induction was positively correlated with both SNIPR binding affinity and antigen abundance (Supplemental Fig. 4F) (*31**,**32*). The induction of luciferase activity also correlated positively with the target antigen abundance (*n* = 4, 1-way ANOVA, *P* < 0.001 for all comparisons of SKBR3) (Supplemental Fig. 4G).

### Activated Anti-HER2-SNIPR T Cells Show Antigen-Dependent [^18^F]FHBG Accumulation In Vitro

Next, we designed SNIPR T cells to secrete IL2 superkine on binding to target antigens (*33*). We created an inducible vector with HSV1-sr39TK and IL2 superkine, linked by T2A self-cleaving peptides (Supplemental Figs. 2B and 5A) (*34*). We induced the anti-HER2 SNIPR T cells by coculturing them with SKBR3 or MD468 cells for 48 h. [^18^F]FHBG was added to the medium, and SNIPR T cells were incubated for 3 more hours (Supplemental Fig. 5B). After removing the supernatant and washing, residual intracellular [^18^F]FHBG was measured using a γ-counter. Anti-HER2 SNIPR T cells cocultured with SKBR3 cells accumulated over 20-fold higher radiotracer levels than did anti-HER2 SNIPR T cells cocultured with MD468 cells (*n* = 3, *P* = 0.013) (Supplemental Fig. 5C). Once again, we tested [^18^F]FHBG radiotracer uptake in suboptimal conditions by using scFvs with lower binding affinities and cancer cells with a lower antigen abundance (*n* = 3 per combination). As expected, [^18^F]FHBG incorporation correlated with both SNIPR binding affinity and target antigen abundance (Supplemental Fig. 5D).

### Imaging Using Anti-HER2 SNIPR PET Shows High Antigen Specificity In Vivo

#### Luciferase-Based Optical Imaging

Since IL2 superkine was not strong enough to induce T-cell survival and proliferation in vivo, we introduced anti-HER2 CAR to the SNIPR T cells to more strongly induce T-cell proliferation and survival as well as reporter (HSV1-sr39TK or fLuc) expression on antigen binding (Supplemental Fig. 6A). The fluorescence-activated cell sorting yield of T-cell transduction with 3 plasmids was in the acceptable range, about 10% (Supplemental Fig. 6B). In this system, anti-HER2 CAR is constitutively expressed but only activates T cells and induces proliferation on binding to its target antigen HER2. As a pilot experiment, we again generated similar-sized SKBR3 (HER2+) and MB468 (HER2–) xenografts by subcutaneous injection, followed by anti-HER2 SNIPR T-cell injection and bioluminescence imaging for the next 21 d (Supplemental Fig. 6C). The luciferase-SNIPR T-cell signal was stronger within the HER2+ tumor, which is maximized at day 9, likely reflecting active proliferation within the tumor microenvironment (145-fold, no *P* value) (Supplemental Figs. 6D and 6E). The signal, however, decreased over time afterward, likely secondary to minimal target-killing activity of CD4 and clearing of target cells (Supplemental Fig. 6F).

#### PET Imaging

**FIGURE 2.**
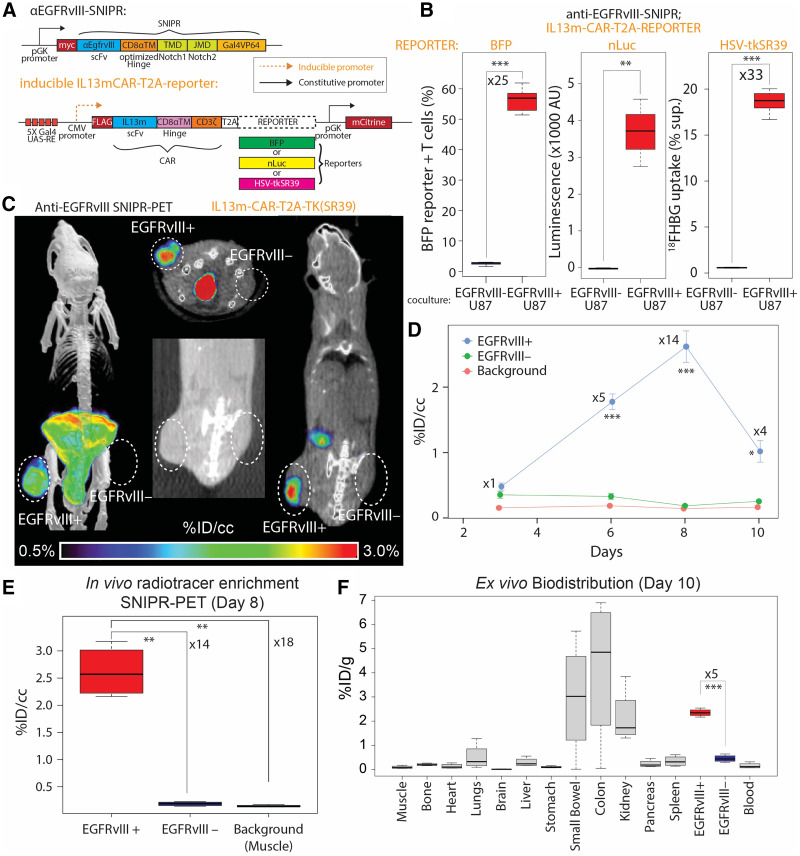
EGFRvIII SNIPR PET. (A) We generated SNIPR T cells with anti-EGFRvIII-SNIPR and inducible IL13-mutein (IL13m)-CAR-T2A-reporter constructs. We used 3 different reporters: BFP, nLuc, and HSV-TKSR39. In this system, SNIPR T cells express anti-EGFRvIII-SNIPR at baseline but do not express IL13m-CAR or reporters. When anti-EGFRvIII binds to EGFRvIII on target cells, SNIPR T cells induce expression of IL13m-CAR and reporters: BFP, nLuc, or HSV-TKSR39. Since most U87 cells express IL13 receptor α-2, T cells expressing IL13m-CAR secrete cytokines and growth factors that induce T-cell proliferation and survival. (B) SNIPR T cells incubated with EGFRvIII+ U87 cells demonstrated significantly higher level of BFP reporter expression, nLuc enzymatic activity, and HSV-TKSR39–mediated ^18^FHBG accumulation than did SNIPR T cells incubated with EGFRvIII– U87 cells. (C) Following a protocol similar to that used for HER2, EGFRvIII+ U87 and EGFRvIII– U87 cells were implanted into mouse flank subcutaneous tissues. At 4 wk after implantation, anti-EGFRvIII T cells with inducible anti-IL13-mutein-CAR-T2A-HSV-TK(SR39) were injected into tail veins. Representative maximum-intensity-projection [^18^F]FHBG PET/CT image (left) and cross-sectional [^18^F]FHBG PET/CT images (middle and right) at day 8 demonstrated high radiotracer enrichment within EGFRvIII+ U87 xenograft compared with EGFRvIII– U87 xenograft on contralateral side. (D) Time-dependent ROI analysis of radiotracer enrichment within EGFRvIII+ xenograft demonstrated greatest radiotracer enrichment at day 8 after T-cell injection, followed by slight decrease in PET signal at day 10, at which point animals were killed for ex vivo biodistribution analysis. (E) Quantitative ROI analysis of EGFRvIII+ and EGFRvIII– tumors and background (shoulder muscle) demonstrated statistically significant radiotracer enrichment within EGFRvIII+ xenograft, 14 times and 18 times greater than within EGFRvIII– xenograft and background. (F) Ex vivo analysis (day 10) of [^18^F]FHBG enrichment within different organs demonstrated significantly greater [^18^F]FHBG enrichment within EGFRvIII+ xenograft than within EGFRvIII– xenograft. As seen on small-animal PET/CT images, gastrointestinal system demonstrated high level of [^18^F]FHBG. *P < 0.05. **P < 0.01. ***P < 0.001.

On the basis of the luciferase data, we generated SNIPR T cells with constitutively expressed anti-HER2 SNIPR and anti-HER2 CAR and with conditionally expressed HSV-sr38TK-T2A-sIL2 (Fig. 1A). We first tested the efficacy of in vitro [^18^F]FHBG uptake on SNIPR activation in the SNIPR-CAR system, following the same experimental scheme as for the SNIPR-only system in Supplemental Figure 5B. As expected, when induced by coculturing with SKBR3 (HER2+) cells, the SNIPR T cells accumulated a 27-times higher amount of [^18^F]FHBG than the SNIPR T cells cocultured with MD468 (HER2–) cells (*n* = 8, *P* < 0.001) (Fig. 1B). We also chose day 8 to image the SNIPR T cells with anti-HER2 CAR and inducible HSV1-sr39TK-T2A-sIL2 in the HER2+/HER2− xenograft model (Fig. 1C). Again, we generated similar-sized HER2+ (SKBR3) and HER2– (MB468) xenografts by subcutaneous injection into NCG mice, 4 wk before the injection of SNIPR-CAR T cells with HSV-sr38TK-T2A-sIL2 reporter (*n* = 7). [^18^F]FHBG imaging was performed at 3, 6, 8, and 10 d by microPET/CT (mPET/CT) (*35**,**36*). Greater [^18^F]FHBG uptake on the HER2+ side was observed than on the HER2− side, indicating that the T cells localized around the HER2+ xenograft (*n* = 7) (Figs. 1D and 1E). There was an approximately 10-fold higher [^18^F]FHBG uptake in the HER2+ xenograft than in the HER2− xenograft (*P* < 0.001), and there was approximately 13-fold higher [^18^F]FHBG uptake in the HER2+ xenograft than in the shoulder muscle (*P* < 0.001), based on region-of-interest (ROI) analysis. In contrast, there was no significant difference in [^18^F]FHBG signal between HER2-xenograft and background muscle (*P* > 0.05). As seen in the SNIPR-CAR system with luciferase reporter in Supplemental Figure 5E, [^18^F]FHBG signal decreased 10 d after T-cell intravenous injection, at which time the tumors were harvested for ex vivo biodistribution analysis (Fig. 1F). The difference in PET signal between HER2+ and HER2– xenografts was 5-fold, with a *P* value of 0.003 at day 10, and the difference in ex vivo radioactivity between HER2+ and HER2– xenografts was 4.2-fold, with a *P* value of 0.036 (Figs. 1F and 1G). Images from the PET study showed marked radiotracer in the stomach, intestine, and gallbladder, consistent with the known biodistribution of [^18^F]FHBG in wild-type animals due to hepatobiliary excretion, as shown in both humans and rodents (Fig. 2D) (*37*–*39*). This result was corroborated using tissue extraction and ex vivo γ-counting at day 10 (Fig. 1G).

### Comparing Anti-HER2 SNIPR PET with Anti-HER2 [^89^Zr]Trastuzumab and [^18^F]FDG

**FIGURE 3.**
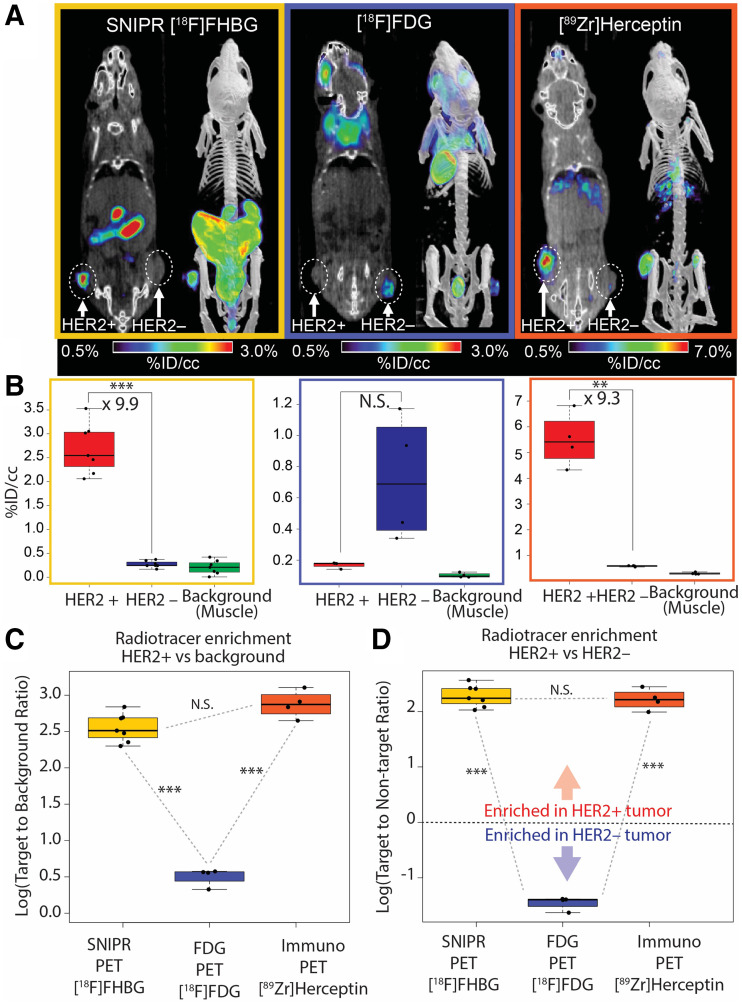
Comparison among SNIPR PET, [^18^F]FDG PET, and immuno-PET. (A) Representative images of [^18^F]FHBG SNIPR PET, [^18^F]FDG PET, and [^89^Zr]trastuzumab PET. SNIPR PET and trastuzumab immuno-PET demonstrated radiotracer enrichment within SKBR3 (HER2+) xenograft compared with MD468 (HER2–) tumor, whereas [^18^F]FDG PET demonstrated higher radiotracer enrichment within MD468 tumor. (B) ROI analysis demonstrated statistically significant, 9.9-fold greater enrichment of [^18^F]FHBG within HER2+ tumor than within HER2– tumor (left), nonstatistically significant enrichment (*P* > 0.05) of [^18^F]FDG within HER2– tumor compared with HER2+ tumor, and statistically significant 9.3 times greater enrichment of [^89^Zr]trastuzumab within HER2+ tumor than within HER2– tumor. (C and D) Fold enrichment of radiotracer within HER2+ tumor was significantly greater in SNIPR PET and immuno-PET than in [^18^F]FDG PET, when compared with background (C) and when compared with HER2– tumor (D). NS = not statistically significant. **P < 0.01. ***P < 0.001.

Trastuzumab has the same anti-HER2 scFv binding moiety as our SNIPR, thereby reflecting the affinity-based interaction of the same antigen–antibody pair (*40*). We used [^89^Zr]trastuzumab (anti-HER2) PET imaging and [^18^F]FDG PET in the same animal model as for the SNIPR-CAR system (*n* = 4). Overall, different biodistributions of the 2 tracers were observed, consistent with distinct metabolism and excretion pathways (Fig. 3A). Both immuno-PET with [^89^Zr]trastuzumab and SNIPR PET with [^18^F]FHBG demonstrated statistically significant increased radiotracer enrichment in HER2+ tumor compared with HER2– tumor (9.9-fold, with *P* < 0.001, and 9.3-fold, with *P* = 0.002, respectively) (Fig. 3B). The relative radiotracer enrichment within HER2+ tumor compared with HER2− tumor was not statistically significant between immuno-PET and SNIPR PET (*P* > 0.05) (Fig. 3C). Likewise, the relative radiotracer enrichment within HER2+ tumor compared with background was also not statistically significant between immuno-PET and SNIPR PET (*P* > 0.05) (Fig. 2D). Imaging results using [^89^Zr]trastuzumab were corroborated via ex vivo analysis of harvested tissues (Supplemental Fig. 7). Although not statistically significant, the trend of higher [^18^F]FDG accumulation in HER2– tumor than in HER2+ tumor on a %ID/cm^3^ basis correlated with the higher growth rate of MD468 (HER2–) than of SKBR3 (HER2+) that we observed both in vitro and in vivo (Figs. 3B and 3D).

### SNIPR PET Can Be Extended to the Glioblastoma Antigen EGFRvIII

To demonstrate the feasibility of SNIPR PET in other cancers, we chose the glioblastoma-specific antigen EGFRvIII. We designed T cells to constitutively express SNIPR that targets EGFRvIII and to conditionally express CAR that targets the distinct antigen IL13 receptor α-2, analogous to our previous approach to targeting glioblastoma with cytotoxic CD8 T cells (*12*). At baseline, those T cells express only anti-EGFRvIII SNIPR, without CAR or reporter. When they recognize EGFRvIII, they express IL13-mutein (IL13m)-CAR, which strongly binds to the more widely expressed but less specific target IL13 receptor α-2, as well as reporter genes (Fig. 2A). To demonstrate in vitro reporter activation on anti-EGFRvIII SNIPR activation, we generated 3 different T cells with blue fluorescent protein (BFP), nano-luciferase (nLuc), and TKSR39 reporters. Those reporters were coexpressed with IL13m-CAR and then cleaved at the intervening T2A sequence to generate separate CAR and reporter proteins. As expected, T cells bearing anti-EGFRvIII SNIPR receptor induced a significantly increased level of BFP and nLuc activity 48 h after coculturing with EGFRvIII+ U87 cells, compared with coculturing with EGFRvIII– U87 cells (BFP: *n* = 4, 25-fold, *P* < 0.001; nLuc: *n* = 4, *P* = 0.002) (Fig. 2B). T cells bearing anti-EGFRvIII SNIPR receptor and inducible IL13m-CAR-T2A-TKSR39 demonstrated a significantly increased level of [^18^F]FHBG uptake when cocultured with EGFRvIII+ U87 cells compared with the same T cells cocultured with EGFRvIII– U87 cells (*n* = 8, 32.5-fold, *P* < 0.001). To demonstrate the potential for in vivo imaging, we again generated a mouse model with EGFRvIII+ U87 and EGFRvIII– U87 xenografts by subcutaneous injection, followed by SNIPR T-cell injection and PET imaging for the next 10 d (*n* = 4) (Fig. 2C). ROI analysis demonstrated a significantly higher level of radiotracer within the EGFRvIII+ xenograft than within the EGFRvIII– xenograft, and this increase was maximized at day 8, based on per-volume radioactivity (%ID/cm^3^) (14-fold, *P* < 0.001) (Figs. 2D and 2E). As seen with the HER2 SNIPR-CAR system, the EGFRvIII SNIPR CAR system demonstrated a decrease in PET signal at day 10, likely secondary to low target killing activity of activated CD4+ T cells (Fig. 2D). Again, EGFRvIII+ and EGFRvIII− xenograft mice were killed at day 10 for ex vivo analysis, which confirmed significantly higher enrichment of radiotracer within the EGFRvIII+ xenograft than within the EGFRvIII– xenograft based on per-weight radioactivity (%ID/g) (5-fold excess, *P* < 0.001) (Fig. 2F).

## DISCUSSION

We have developed an antigen-specific, PET-compatible SNIPR T-cell reporter system in response to the rapidly increasing interest in T-cell–mediated treatment of human tumors. This customizable synthetic receptor platform can provide an essential companion to CAR T–mediated treatment, in addition to the recent application of synNotch to oncologic challenges (*12*). Applying several rounds of synthetic biology, we engineered SNIPR T cells that are antigen-inducible, as detected by GFP fluorescence, luciferase luminescence, and the HSV-TK PET reporter [^18^F]FHBG, and can successfully be imaged in dual-xenograft animal models. We have demonstrated application of this imaging modality to 2 different tumor antigens: HER2 and EGFRvIII. This technology might be used as a companion biomarker for CAR T therapies, such as to characterize off-target effects or verify tumor engagement. As the technology matures, SNIPR PET might be used to detect and characterize molecular profiles of early cancers without biopsy.

## CONCLUSION

We have developed an antigen-inducible T cell PET reporter system using the versatile SNIPR. This reporter system may be used in a variety of T cell treatments for imaging antigen engagement.

## Notes

KEY POINTS **QUESTION:** Can an inducible, cell-based PET system image tumor antigens in vivo? **PERTINENT FINDINGS:** An inducible PET reporter system can be coexpressed with CAR in therapeutic T cells to image HER2 and EGFRvIII. **IMPLICATIONS FOR PATIENT CARE:** As cell-based therapies mature, SNIPR PET might be added to any therapeutic T cell to image antigen engagement.
